# Remarks on the Nature and Cause of Cancerous Disease, and the Indications for Its Cure

**Published:** 1852-05

**Authors:** H. A. Johnson

**Affiliations:** Chicago


### THE NORTH-WESTERN

### MEDICAL AND SURGICAL JOURNAL.

NEW SERIES.

VOL. I. MAY, 1852. No. 1.

### ORIGINAL COMMUNICATIONS.

ARTICLE I.—*Remarks on the Nature and Cause of Cancerous Disease, and the Indications for its Cure.* By H. A. JOHNSON, M. D.

A CANCEROUS tumor consists of two elements, a stromal and a cellular; the first appearing to be formed of cells brought forcibly in contact and retained there until absorption of their walls takes place, while the nuclei remain, thus presenting the distinctly fibrous character of the earlier stages of the disease. The second element, consisting of free cells and granules, seems to result from an arrest of growth, and a disintegration of the fibrous structure, and perhaps dependent on the removal of pressure from the surrounding parts, as it always predominates in the second or soft stage. The cells differ from those of healthy growths and non-malignant tumors in their increased size, in their comparatively large nuclei, and in their rapid production and disintegration. So far we are carried by the microscope, which, while it has revealed very faithfully the structural pathology, has suggested no rational method for the cure of this disease. The instrument, however, is not at fault. A mass of unused facts has been accumulated which, when looked at by the light of other truth, may lose their amorphic character, and be transformed, if not into perfect crystals, into shapes at least intelligible.

A chemical examination of malignant tumors shows a very great departure from the constitution of healthy tissues. The following analysis of Scirrhus is by Foy:

| | |
|---|---|
| Albumen, fatty matter, fibrin, and water, - | 61.10 |
| Iron, - - - - - - - | 1.65 |
| Carb. soda, - - - - - - - | 5.00 |
| Bone phos. lime, - - - - - | 16.60 |
| Carb. of lime, - - - - - - | 6.60 |
| Other salts, - - - - - - | 8.95 |

The Encephaloid variety, according to the same authority, gave:

| | |
|---|---|
| Albumen, fat, fibrin, water, &c., - - | 78.35 |
| Oxyd of iron, - - - - - - | 1.35 |
| Carb. soda, - - - - - - | 2.75 |
| Bone phos. lime, - - - - - | 6.30 |
| Carb. of lime, - - - - - - | 4.00 |
| Other salts, - - - - - - | 5.95 |

The relation of the organic to the inorganic constituents in healthy tissues varies according to the structure, thus rendering even an approximate comparison difficult, and an accurate one impossible; but if those parts most frequently the seat of the disease be taken as the standard, we find that in malignant growths the salts, and especially those of lime, are in very great excess, while the protien compounds are relatively deficient.

The location of the disease in glands, or on surfaces performing the function of glands,—as the skin, mucous membrane of the stomach, &c., all the seats of abundant cell growths—taken in connection with its structure, seems to indicate as the first step in the departure from the healthy performance of the functions of those organs, the production, instead of a healthy, of a morbid cell, the pathology of which consists in its special characters as before noticed; while the relatively large amount of the inorganic substances sustains to the cell structure the relation either of cause or effect.

The rapid production and disintegration of cell structures would, by oxydation of the phosphorus of the albumen, result in an excess, not only in the excretions, but in the system and especially in the diseased part, of the phosphatic salts, and in this manner they may be regarded as a result of the disease.

On the other hand, we may suppose that after the complete development of the osseous system, there being very much less demand for calcareous substances, they accumulate in excess, and after having passed the limit of healthy variation, produce sensible disease; at first local in its character, but very soon, especially if situated in parts where absorption is active, becoming constitutional and giving rise to the peculiar cachexia.

The local affection being once established, the excess of the inorganic substances may be perpetuated or even increased, as already indicated, by the rapid metamorphosis of azotized nutriment, thus giving rise to its malignancy. In this manner we may have the salts both as a cause and as an effect,—an hypothesis which seems to me to be sustained by numerous facts.

It is in accordance with the economy of nature to suppose that the inorganic constituents of the blood, coming as they do in contact with all the tissues, must, even though designed in the main for the bony system, possess an important influence in the nutrition of the soft parts, and that any appreciable change in the quantity of the salts would modify in a corresponding degree the assimilative process.

The frequent location of the disease in the testicle and female breast, parts to which there is a determination of these salts, and the secretions of which contain them in a large per centage, seems to strengthen this hypothesis.

The relation which the disease bears to the condition of the osseous system, being often associated with softening of the bones, together with the fact of its making its appearance more generally in advanced life after the complete development of the skeleton, seems to favor the same idea; that is, that the disease is in some way dependent on the accumulation or morbid distribution of the calcareous and perhaps other inorganic substances. It is true the Encephaloid forms an exception to the rule in regard to age; but, as will be seen by referring to the analysis, the per centage of the salts, though less than in Scirrhus, is yet much above the standard for healthy tissues, and this fact together with the great activity of the digestive and assimilative organs and the consequent copious supply of nutriment, seems to account satisfactorily not only for the existence but for the rapid growth during the earlier periods of life of this variety of the disease.

The circumstances under which the disease originates, and the relation which it bears to other affections, seem to confirm our view of the subject. Chelius says, "it is more common in persons who are very sensitive or melancholic, who lead a sedentary life, and who have suffered much care and trouble," "that its occasional cause is all mischief which produces constant but not intense irritation." These circumstances are all calculated to give rise to the phosphatic diathesis. Scarpa says the disease attacks persons "in whom, if there be suspicion of a diathesis, it is not that of scrofula." The same opinion has been entertained by others, and it is believed by Prof. Stone "that true Cancer never occurred in decidedly scrofulous subjects." From recent observations both in Europe and in this country, it seems probable that scrofula and tubercle are benefited by the use of phosphate of lime. The natural inference is, that if in tuberculosis the phosphates are deficient and require to be exhibited, and if cancerous disease is the opposite, the excess of the salts must be considered as an essential element of that disease, and they require elimination.

With the above facts before us, we are naturally led to enquire what influence the salts, and especially those of lime, soda and potash, are known to possess in the production of structures analogous to those under consideration. The first recorded experiments, so far as I know on this subject, are those of Beneke, who has administered the phosphate of lime internally with, as he believes, the evident effect of promoting cell growth in the system; it has since been used by others with like apparent results. In order to test this substance still farther, the following experiment was entered into by Dr. Beneke: Albumen (of the egg,) mingled with a small quantity of fat and phosphate of lime, was subjected for eight hours to a constant temperature of 104° F., when globules were found containing nuclei, and resembling, in all their characteristics, cells produced in the living body. A mixture of albumen and fat alone, under the same circumstances, failed to give the same results. These experiments have been carefully repeated and varied by the writer. From the notes made at the time the following is selected:

Exp. No. 1. Albumen, fat, and water intimately mingled together. Exposed to a uniform temperature of 104° F.—Examined after nine hours—contains oil globules, granules, and a few imperfect celluloid bodies.—After twenty-two hours no perceptible change noticed.

Exp. No. 2. Albumen, oil, water, and phosphate of lime.—At the expiration of ten hours, contains granules and bodies resembling the cells found in pus and mucus.—After twenty-two hours, granules previously noticed very abundant,—bodies resembling pus or mucus globules, with masses of matter very similar to those sometimes found in the urine and composed of epithelial scales.—After seventy hours, fewer of the granules and a large number of the *celluloid* bodies. Upon the addition of acetic acid dark spots are seen having all the appearance of nuclei; each globule seems to have a perfect cell wall.

Exp. Nos. 3, 4, 5, 6 were variations of Nos. 1 and 2, by subjecting to pressure during the process, and by adding to the preparation a small quantity of super carb. of soda in solution. The results were similar to those previously noticed.

Exp. No. 7. Oil, phosphorus, and water enclosed in a piece of bladder and placed in a cup containing albumen.—After thirty-six hours, the albumen in the cup contains granules and celluloid bodies as in previous experiments,—the bladder is nearly empty with the exception of gas with which it is distended. In the drop of fluid remaining there is found an abundance of the phos. lime, oil globules and the celluloid bodies. Acetic acid affects them precisely as it does organic cells. Ether renders the walls more opaque.

Exp. No. 8. Repetition of No. 7. with like results.

Exp. No. 9. Albumen, water, and super carb. soda in a glass tube, closed by clean, fresh membrane, and immersed in a cup containing oil, water and phos. lime held in solution by acetic acid.—After twenty hours the fluid in the cup contains granules, celluloid bodies, and triangular prismatic crystals almost exactly like those of the triple phosphate found in the urine. In the tube are granules and a few celluloid bodies.

Exp. No. 10. Albumen, water, phosphate of lime, slightly acidulated with acetic acid, in a tube closed by membrane, and placed in a cup containing oil, super carb. soda, and water.—After twenty hours the fluid has all passed out of the tube into the cup, which contains bodies like those already described, and others of greater size, more irregular outline, and containing large, distinct nuclei.

Exp. No. 13. Oil, phos. lime, and water slightly acidulated, in a tube closed by membrane and placed in a cup containing albumen, water and carb. potassa.—After twenty-four hours the cup and tube both contained celluloid bodies of a small size and very regular outline.

The results of these experiments sustain the hypothesis of Dr. Beneke, that the phosphate of lime has an influence in promoting cell growth; and they further show that the size and character of the cell may be modified by other salts, especially those of soda and potash, and also by a variation in the conditions under which the substances are brought in contact,—see experiment No. 10, in which there were cells very similar to those of Cancèr, as will be observed by comparing the two descriptions.

The facts above given, seem to me to indicate very strongly, that the presence in cancerous growths of the inorganic substances in greater proportion than in healthy tissues, is not an accidental circumstance; but, on the contrary, that at least one essential and important element of the disease consists in the excess of these salts, while the characteristic cell is probably produced by their action on the albumen and fat, supplied by the nutritive organs.

If the above inference be correct, we should expect that the growth of malignant tumors would be retarded, if not arrested, by any dietetic or medicinal treatment, calculated to prevent the introduction or favor the elimination of the phosphatic or other salts. Chelius advises that "animal food be avoided, and milk or vegetable diet ordered." Prof. Stone, of New Orleans, in an article entitled "Hints on Cancerous Affections," remarks:

"Experience has shown that the least nitrogenous diet is best in this disease. In the memoirs of the celebrated Nathan Smith, written some twenty years ago by his son, Nathan R. Smith, of Baltimore, are found the views of this remarkable man, which were based purely upon observation, without a chemical idea to theorise upon. His diet for this disease was vegetable; and of this he thought green corn the best. A case is related of a lady on whom he operated for a very large cancerous breast, involving the glands of the axilla. It was in the season for green corn, and the patient was put upon this article of diet. Sufficient was gathered when in the milk, and dried, to last until the season returned, and this made soft by boiling, and used with little or no seasoning. He states that whenever she attempted to return to her usual diet, she experienced shooting pains in the part, but finally, after two years, she gradually changed her diet. The notice of this case was given seven years after the operation, and there was no appearance of a return of the disease. Corn in this state contains, I believe, more phosphorus than any other vegetable, but whether this renders it more suitable to this disease, I am not prepared to say. Professor Bigelow, of Boston, relates a case in which diet kept a cancerous affection dormant, at least, for many years, or rather he states that it was gradually getting well. This was the case of the late distinguished surgeon Amos Twitchell, of Keene, N. H., with whom I was well acquainted. I dined with him in 1848; he furnished a good dinner for his guests, but dined himself on milk and berries. Vegetables of the blandest kind constituted his main food, but I do not think he confined himself to any one article. The disease was seated in the inner canthus of one of his eyes, was removed many years ago with the knife, but the cicatrix soon took on the same degeneration, and he relied upon diet; and although it might appear to a gourmand a very meagre diet, he was able to undergo more fatigue at the age of 64 than many young men. I mention these cases not as being remarkable in themselves, but because they illustrate the effects of diet, and at the same time cover the views of two medical men, remarkable for their powers of observation."

The course of diet recommended above may be beneficial, as it seems to me, for two reasons: First, Vegetable food contains less of the salts than animal. It is evident, therefore, that by such a diet we, partly at least, prevent the introduction into the system of those substances. Secondly, The use of a diet consisting mainly of starch, gum, sugar, fruits, milk, &c., favors the production in the system, of the organic acids, and especially of the lactic, in which the calcareous and other salts are all soluble, and hence are more readily thrown off in the secretions.

The cachexia almost always present in this disease, requires a generous, supporting diet, from which animal food cannot, with safety, be entirely excluded; we are, therefore, compelled to look for *medicinal* agents for the accomplishment of the main object, namely: the elimination of the inorganic substances.

The organic acids, acting as solvents for the saline constituents, seem best adapted for fulfilling the above indication. Lactic acid especially, is known to possess the property of holding in solution a large quantity of the phosphate of lime; if, therefore, it can be introduced into the system in a free state, or in the form of an easily decomposed salt, as for instance the proto salt of iron, we may reasonably expect that it will produce a change in the quality of the circulating fluids.

In order to test the re-action of the lactate of iron, I added gradually to a solution of it a small quantity of bone ash. After a short time, a precipitate of a pale, greenish color fell to the bottom of the glass, and when the fluid had become perfectly clear, it contained none of the iron salt, as was shown by the addition of a small quantity of the prussiate of potash in solution. Oxalic acid produced a copious deposit of oxalate of lime. Is it not probable, therefore, that if this salt be introduced into the circulation, a double decomposition would take place,—the lactic acid uniting with the lime to produce a soluble compound, readily excreted, while the iron, from its tonic properties, might be beneficial in the almost always debilitated condition of the patient?

Whether it acts in the manner thus indicated, or in some other way entirely different, it seems from the observations of Prof. Brainard, reported in the *Am. Journal of Med. Sciences* for April, 1852, that the lactate of iron *does* retard the growth of Cancerous disease. Prof. B. is continuing his observations on the effects of this salt administered both by the stomach and by injections into the veins.

In conclusion, we venture to make the following propositions as probably true:

1st. That the essential element of Cancerous disease is a morbid accumulation, or distribution, of the calcareous and other salts.

2d. That the morbid accumulation or distribution of the salts, furnishes the conditions necessary for the development of the cellular and fibrous structure.

3d. That the indications for the cure of Cancer are, the removal from the system and an exclusion, as far as possible, of any excess of the saline substances.

4th. That, in the present state of our knowledge, the means best adapted for fulfilling these indications are, a regulated diet, consisting of animal food—flesh and milk,—combined with starch, sugar, fruits, &c., in large proportion; and the administration, both locally and generally, by the stomach and veins, of the organic acids, and especially the lactic, feebly combined with non-alkaline bases.

CHICAGO, April 20th, 1852.

